# Association Between Serum Symmetric Dimethylarginine and Tooth Resorption in Cats: An Exploratory Study on the Oral-Renal Axis

**DOI:** 10.3390/vetsci13030233

**Published:** 2026-02-28

**Authors:** Kurtuluş Parlak, Murat Kaan Durgut, Hilmican Ergin, Elgin Orçum Uzunlu, Selman Pulat, Furkan Çağrı Beşoluk

**Affiliations:** 1 Department of Surgery, Faculty of Veterinary Medicine, Selcuk University, Selçuklu, Konya 42130, Türkiye; hilmican.ergin@selcuk.edu.tr (H.E.); selmanpulatt54@gmail.com (S.P.); 2 Department of Internal Medicine, Faculty of Veterinary Medicine, Selcuk University, Selçuklu, Konya 42130, Türkiye; mkaan.durgut@selcuk.edu.tr; 3 Department of Wild Animal Diseases and Ecology, Faculty of Veterinary Medicine, Dicle University, Sur, Diyarbakir 21280, Türkiye; elginorcum.uzunlu@dicle.edu.tr; 4 Department of Veterinary Biostatistics, Faculty of Veterinary Medicine, Selcuk University, Selçuklu, Konya 42130, Türkiye; furkan.besoluk@selcuk.edu.tr

**Keywords:** feline tooth resorption, SDMA, oral-renal axis, systemic inflammation

## Abstract

Background: Tooth resorption (TR) is a prevalent dental condition with under-investigated systemic implications, particularly regarding renal health. This study aimed to evaluate renal biomarkers, specifically symmetric dimethylarginine (SDMA), and the association between TR and blood parameters. Methods: Thirty-six cats (24 TR; 12 controls) were evaluated via intraoral radiography and blood analysis. Multivariable logistic regression was performed to identify independent predictors for TR, adjusting for age and sex. Results: Tooth resorption was diagnosed in 66.7% of the cats, predominantly affecting the mandibular first molars (409) and fourth premolars (108). A strong association was identified between TR stages and lesion types (*p* < 0.001). Cats with TR exhibited significantly higher serum SDMA concentrations (*p* = 0.011) and significantly lower albumin levels (*p* < 0.001) compared to controls. Hematological analysis revealed marked leukocytosis and monocytosis (*p* < 0.001) in the TR group. Most importantly, multivariable logistic regression identified SDMA as a predictor with TR presence (*p* = 0.012), with each unit increase in SDMA associated with a 3.5-fold increase in the likelihood of having resorptive lesions, independent of age and sex. Conclusions: Our findings regarding the oral-renal axis emphasize that feline TR involves chronic systemic inflammation. Consequently, it should be managed as a condition with measurable systemic impacts rather than merely a localized disease.

## Simple Summary

Tooth resorption is a prevalent and painful dental condition in cats characterized by the breakdown of tooth structure. While currently treated primarily as a localized oral disease, its potential impact on the cat’s overall health, particularly kidney function, has not been fully explored. This study aimed to evaluate the relationship between tooth resorption and kidney health by measuring serum symmetric dimethylarginine, a sensitive biomarker for early kidney function decline. We analyzed this association in a group of cats, carefully balancing for age and sex to ensure accurate results. The study indicates a significant connection between oral health and renal function, suggesting that cats with tooth resorption experience chronic systemic inflammation. We conclude that tooth resorption should be viewed not merely as a dental issue but as a condition with measurable effects on the whole body. These findings are valuable for cat owners and veterinarians as they highlight the importance of monitoring kidney health in feline dental patients to ensure comprehensive medical care.

## 1. Introduction

Tooth resorption (TR) in cats is a common and clinically important dental condition characterized by the progressive loss of mineralized dental tissues of permanent teeth due to odontoclastic activity [1,2,3]. The condition has been described using various terms, including feline odontoclastic resorptive lesions and resorptive lesions, reflecting the persistent uncertainty regarding its etiology [1,3,4,5]. Currently, “tooth resorption (TR)” is the terminology endorsed by the American Veterinary Dental College (AVDC) and is widely accepted in veterinary dentistry [6].

Several hypotheses have been proposed to explain the pathogenesis of TR, including genetic predisposition, dietary factors, and inflammatory mechanisms; however, none has been conclusively demonstrated [7,8,9,10,11,12]. Although TR frequently occurs alongside periodontal disease, no consistent clinical, radiographic, or histopathological association between these conditions has been established, suggesting that TR represents a distinct pathological process [7,8,9,10,11,12].

Despite its high prevalence and progressive nature, research investigating potential systemic associations of TR in cats remains limited. Most available studies have focused primarily on local oral pathology, while possible extraoral or systemic alterations accompanying TR have received comparatively little attention. Reiter et al. [9] reported lower urine specific gravity in cats affected by TR, whereas Whyte et al. [13] described changes in selected blood parameters, including reduced albumin-to-globulin ratios, which may reflect systemic inflammatory responses. These findings suggest that TR may coexist with measurable systemic alterations; however, the nature and clinical relevance of these associations remain unclear.

Symmetric dimethylarginine (SDMA) is a sensitive biomarker of glomerular filtration rate (GFR) and has been shown to increase earlier than serum creatinine in cats and dogs with declining renal function [14]. Owing to its diagnostic value, SDMA has been incorporated into the International Renal Interest Society (IRIS) guidelines for the staging of chronic kidney disease (CKD) in companion animals. Nevertheless, data regarding SDMA concentrations in cats affected by TR are scarce. While the association between general periodontal disease and renal dysfunction is documented in feline medicine, the specific predictive value of SDMA in cats with tooth resorption independent of common clinical confounders remains to be fully elucidated. This study aims to address this gap by evaluating the relationship between TR and SDMA through a multivariable logistic regression model, thereby investigating the potential existence of a distinct oral-renal axis associated specifically with resorptive lesions. While tooth resorption (TR) frequently co-occurs with periodontal disease, it is increasingly recognized as a distinct pathological entity characterized by idiopathic, progressive odontoclastic destruction of mineralized dental tissues, rather than the bacteria-driven inflammatory breakdown of the periodontium. Unlike generalized periodontal disease, the unique localized tissue turnover and chronic nature of active resorptive lesions may trigger a specific, low-grade systemic inflammatory response [15]. Our mechanistic hypothesis suggests that this persistent inflammatory milieu could lead to the release of pro-inflammatory mediators into systemic circulation, potentially influencing renal microcirculation or cellular arginine metabolism [15,16]. It is suggested that these pathways may be associated with alterations in early-stage renal biomarkers like SDMA; however, the primary focus of this study is to explore these clinical associations rather than to establish definitive causal mechanisms. While the oral-renal axis is a well-established conceptual framework in feline periodontal disease, its specific extension to tooth resorption is relatively novel and requires a more nuanced exploration. Since TR involves distinct odontoclastic activity and localized chronic inflammation, it may contribute to the systemic inflammatory load through different pathways than those of primary periodontitis [17]. Therefore, the present investigation should be regarded as an exploratory step in evaluating whether resorptive lesions independent of established periodontal status are linked to alterations in renal biomarkers like SDMA.

Accordingly, the primary objective of the present study was to evaluate the association between serum symmetric dimethylarginine (SDMA) concentrations and tooth resorption (TR) in a population of cats meticulously balanced for age and sex to mitigate potential confounding variables. Secondary objectives included a comprehensive evaluation of selected biochemical and hematological parameters, specifically blood urea nitrogen, creatinine, phosphorus, total protein, and albumin to characterize the systemic alterations and inflammatory markers accompanying tooth resorption. We hypothesized that cats affected by TR would exhibit significant elevations in SDMA and alterations in systemic inflammatory profiles compared to age-matched healthy controls, potentially reflecting a link between chronic oral pathology and systemic biomarkers.

## 2. Materials and Methods

### 2.1. Animal Selection and Inclusion Criteria

This study was designed as a prospective, observational case–control study. Patient recruitment and data collection were conducted over a two-year period at a veterinary teaching hospital. All clinical, radiographic, and laboratory data were collected synchronously during the recruitment period following a standardized research protocol established after institutional ethics committee approval. The study population consisted of 36 client-owned cats presented for dental evaluation or elective surgical procedures. The study population was divided into two groups: the tooth resorption group (TR; *n* = 24) and the control group (*n* = 12), which showed no clinical or radiographic evidence of TR (Table 1). While clinical signs such as halitosis, hypersalivation, and gingivitis were noted in the TR group, the periodontal status of all cats was objectively assessed according to the AVDC (American Veterinary Dental College) Periodontal Disease Staging System (Stages 0–4). In an effort to differentiate the potential systemic associations of tooth resorption from those of generalized periodontal inflammation and to mitigate residual confounding, the study aimed to include only cats classified as Stage 0 (Healthy) or Stage 1 (Gingivitis without attachment loss). Cats exhibiting radiographic evidence of alveolar bone loss or significant attachment loss (Stages 2, 3, and 4) were excluded from the primary analysis. This inclusion threshold was utilized to better characterize the observed systemic alterations within the context of resorptive pathology rather than general periodontal inflammation. Diagnosis of TR was established through a comprehensive clinical oral examination and full-mouth intraoral dental radiography. To maintain diagnostic consistency, oral examinations were performed by a single experienced investigator, and periodontal health was objectively staged according to the AVDC system (Stages 0–4). Similarly, full-mouth radiographs were independently reviewed by two investigators based on AVDC guidelines (Types 1–3; Stages 1–5), with consensus reached through structured discussion in cases of initial disagreement. Regarding laboratory assessments, internal quality was maintained through daily routine calibrations performed at the animal hospital laboratory [6]. The control group comprised cats presented for routine health checks or elective surgeries (e.g., orchiectomy). To ensure the complete absence of resorptive lesions, particularly occult Type 2 lesions that are not detectable by visual inspection, all cats in the control group also underwent full-mouth intraoral digital radiography following the same standardized protocol as the TR group. To ensure a robust comparison and mitigate the confounding effects of age, an age-balanced control group was utilized, ensuring no statistically significant difference in age existed between the groups (*p* > 0.05). Written informed consent was obtained from all owners prior to inclusion. Exclusion criteria included a history or clinical evidence of chronic systemic disease, oral neoplasia, trauma-related dental fractures, or treatment with antibiotics, corticosteroids, or nonsteroidal anti-inflammatory drugs (NSAIDs) within the preceding three months. Only cats older than one year were eligible for inclusion. To ensure a healthy baseline renal status and to exclude cats with overt or subclinical renal disease, a comprehensive screening was performed. Cats were required to have no history of polyuria or polydipsia, normal kidney palpation during physical examination, and serum creatinine concentrations within the laboratory’s established reference ranges.

The median age of cats in the TR group was 3.33 years, while the median age in the control group was 2.83 years. Sex distribution within the groups was as follows: 12 males and 12 females in the TR group, and 11 males and 1 female in the control group. Regarding breed distribution, Domestic Shorthair cats predominated in the TR group (54.16%), whereas in the control group, Domestic Shorthair and Scottish Fold breeds were equally represented, each accounting for 33.3% of the group. Feeding practices were consistent across both groups, with mixed diets reported in 58.33% of the TR cats and 50% of the control cats. To ensure a robust comparison and minimize the potential confounding effects of age, an age-balanced control group was utilized for this study (*p* > 0.05 for age between groups). Although the clinical nature of case recruitment is acknowledged, this alignment facilitates a more reliable interpretation of the identified associations between tooth resorption and systemic biomarkers. Statistical comparisons were primarily directed toward identifying independent associations rather than establishing direct causal relationships.

### 2.2. Preanesthetic Evaluation, Clinical and Radiographic Dental Examination

All cats underwent a standardized preanesthetic evaluation prior to dental examination, radiographic imaging, or elective surgical procedures. This evaluation included a physical examination with assessment of vital parameters, alongside routine hematological and biochemical analyses. Blood gas analysis was performed using a GEM Premier 3000 analyzer (Instrumentation Laboratory, Bedford, MA, USA), complete blood counts were obtained using an MS4e analyzer (Melet Schloesing Laboratories, Osny, France), and serum biochemical analyses were conducted using a BT 300 analyzer (Biotecnica Instruments, Rome, Italy). These assessments were utilized to identify any contraindications to general anesthesia and to ensure maximum patient safety.

Preanesthetic medication consisted of butorphanol hydrogen tartrate (0.1 mg/kg, IM; Butomidor^®^, Richter Pharma, Wels, Austria) and medetomidine hydrochloride (0.025 mg/kg, IM; Domitor^®^, Zoetis, Florham Park, NJ, USA). General anesthesia was induced with intravenous propofol administered to effect (1.5–3 mg/kg; Propofol-Lipuro^®^ 1%, B. Braun Melsungen AG, Melsungen, Germany). Following induction, all cats undergoing surgical procedures were endotracheally intubated to protect the airway against aspiration and to ensure the maintenance of anesthesia through the stable delivery of inhalant agents. Anesthesia was maintained with isoflurane (Isoflo^®^, Zoetis, Florham Park, NJ, USA) delivered in 100% oxygen via a precision vaporizer. To uphold ethical standards of pain management, procedure-specific local anesthesia was administered for all invasive interventions. For cats requiring dental extractions or crown amputations, local anesthesia via infraorbital or mandibular nerve blocks was performed. For cats in the control group undergoing elective orchiectomy, local anesthesia was achieved via spermatic cord block. Postoperative analgesia for all surgical cases was ensured with meloxicam (0.1 mg/kg orally on the first day, followed by 0.05 mg/kg once daily for three consecutive days). This comprehensive protocol was standardized across groups to minimize variability related to drug selection and physiological effects.

**Figure 1:**
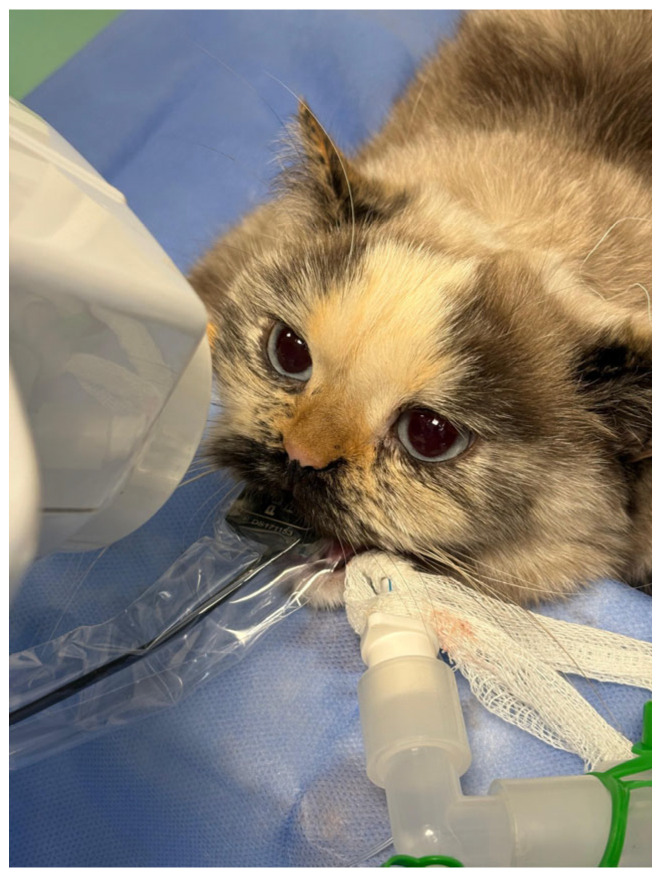
Dental radiography procedure in the cat.

Intraoral dental radiographs were obtained using a Planmeca ProX periapical X-ray unit (Planmeca Oy, Helsinki), Finland, a Planmeca ProSensor RVG (size 1) (Planmeca Oy, Helsinki), and Planmeca Romexis software (version 6.0; Planmeca Oy, Helsinki). Full-mouth radiographic examinations were performed using the parallel and bisecting angle techniques (Figure 1) [2,17,18]. Radiographic images were independently evaluated by two experienced veterinary dentists according to the American Veterinary Dental College (AVDC) classification system. In cases of disagreement, a third evaluator was consulted to achieve consensus. Tooth resorption type and stage were recorded using standardized examination forms [6].

### 2.3. Sampling and Laboratory Analysis

From each cat, 3 mL of blood was collected from the cephalic vein into serum tubes without anticoagulant. To ensure that biochemical parameters were not influenced by the potential effects of anesthetic or sedative agents on glomerular filtration rate (GFR), all blood samples were obtained following a 12 h fast and prior to the administration of any anesthetic or sedative agents. Samples were allowed to clot for 15 min at room temperature and centrifuged at 2000× *g* for 10 min. Serum SDMA concentrations were measured within 45 min of collection using an IDEXX Catalyst One analyzer (IDEXX Laboratories, Westbrook, ME, USA), employing an enzymatic colorimetric dry-slide method validated against LC–MS/MS. All analyses were performed according to the manufacturer’s instructions using non-hemolyzed serum.

### 2.4. Statistical Analysis

Statistical analyses were performed using IBM SPSS Statistics for Windows, version 26.0 (IBM Corp., Armonk, NY, USA). Data distribution was assessed using the Shapiro–Wilk test; as the data were not normally distributed (*p* < 0.05), non-parametric tests were applied. Age and sex distributions between the TR and control groups were compared using the Mann–Whitney U test and the chi-square test, respectively, to verify group homogeneity. Continuous variables were expressed as median (minimum–maximum) values. Effect sizes for univariate comparisons were calculated as r = |Z|/√N, where Z is the standardized Mann–Whitney test statistic and N is the total sample size [18]. Effect sizes were interpreted according to Cohen’s guidelines: r < 0.10 (negligible), 0.10–0.30 (small), 0.30–0.50 (medium), and ≥0.50 (large) [19]. No correction for multiple comparisons was applied across the 47 parameters assessed in Table 4, given the exploratory nature of the present study; findings should be interpreted with appropriate caution and considered hypothesis-generating rather than confirmatory. A binomial logistic regression model (Enter method) was constructed to identify independent predictors for the presence of TR. To ensure model parsimony and avoid multicollinearity among highly correlated renal biomarkers, variables were selected based on their clinical relevance and univariate significance (*p* < 0.20). Blood urea nitrogen (BUN) was excluded from the multivariate model due to its high correlation with other renal parameters and lack of univariate significance. The final multivariate model included SDMA, creatinine, age, and sex as independent variables. Multicollinearity among predictor variables was assessed using Variance Inflation Factors (VIF) obtained from a preliminary linear regression analysis, with VIF < 5 considered acceptable [20]. All predictor variables demonstrated acceptable collinearity (VIF range: 1.007–2.875; tolerance range: 0.348–0.993), indicating that multicollinearity did not compromise model stability. Odds ratios (ORs) and 95% confidence intervals (CIs) were calculated to evaluate the strength of these associations. Post hoc power analyses were conducted using G*Power software (version 3.1.9.7; Heinrich Heine University Düsseldorf, Düsseldorf, Germany) to evaluate the adequacy of sample size for detecting observed effects [21]. For SDMA, the primary outcome of interest, power was calculated for both univariate comparison (Mann–Whitney U test) and as a logistic regression predictor, using observed effect sizes and α = 0.05. Power was additionally calculated for all remaining logistic regression predictors and for the chi-square test of sex distribution. A power of ≥0.80 was considered adequate for robust statistical inference. Model fit was assessed using the Hosmer–Lemeshow goodness-of-fit test. The ratio of events per variable (EPV) was calculated as the number of outcome events divided by the number of predictors in the final model.

## 3. Results

### 3.1. Demographic Characteristics and Clinical Distribution of Lesions

**Figure 2:**
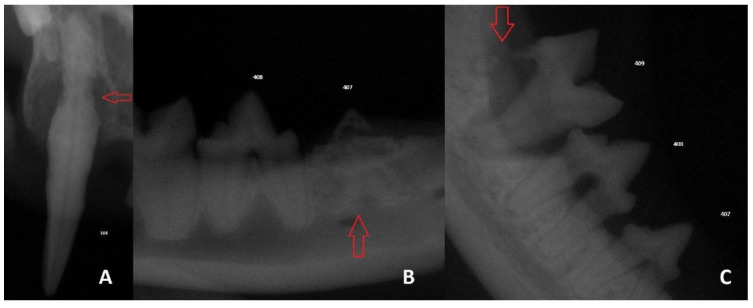
Radiographic examination images of the TR group cats. The red arrows indicate the areas of tooth resorption (**A**) Stage 2 and Type 1 resorption in tooth 104; (**B**) Stage 5 and Type 3 resorption in tooth 407; (**C**) Stage 4c and Type 3 resorption in tooth 409.

The study population comprised 36 cats, including a tooth resorption (TR) group (*n* = 24) and a control group (*n* = 12). Demographic analysis confirmed that the groups were well-balanced regarding age, with a median age of 3.33 years for the TR group and 2.83 years for the control group (*p* > 0.05). While a higher proportion of males was noted in the control group (11 males, 1 female) compared to the TR group (12 males, 12 females), multivariate modeling demonstrated that sex was not an independent predictor of TR presence (*p* = 0.276). In terms of breed distribution, Domestic Shorthair cats were predominant in the TR group (54.16%), whereas Domestic Shorthair and Scottish Fold breeds were equally represented in the control group (33.3% each). Clinical examinations of the affected cats frequently revealed gingival hyperplasia, halitosis, and hypersalivation. A total of 118 teeth with resorptive lesions were identified in the TR group. As illustrated in Figure 2 and Table 2, the most frequently affected teeth were the mandibular right first molar (tooth 409; 13.56%), followed by the maxillary right fourth premolar (tooth 108; 9.32%), the mandibular left first molar (tooth 309; 8.47%), and the maxillary left fourth premolar (tooth 208; 7.63%).

### 3.2. Association Between Resorption Stage and Type

A chi-square analysis was conducted to assess the relationship between the progression stage and the radiographic type of resorption based on the total number of affected teeth. The analysis revealed a statistically significant association (chi-square = 75.491; df = 12; *p* < 0.001) with a moderate strength of association (Cramér’s V = 0.543). The distribution patterns, presented in Table 3, indicate distinct pathological trends: early stage lesions (Stages 1 and 2): Predominantly associated with Type 1 resorption. Advanced-stage lesions (Stages 3, 4, and 5): More frequently associated with Type 3 resorption.

### 3.3. Biochemical and Hematological Alterations

Comparisons between the TR and control groups demonstrated extensive systemic involvement, characterized by significant differences in biochemical and hematological parameters (Table 4). Effect size analysis revealed substantial variation in the magnitude of differences across parameters. The largest effect sizes were observed for erythrocyte and platelet indices, including RDW (r = 0.710), platelet count (r = 0.710), and plateletcrit (r = 0.699), followed by MCHC (r = 0.668) and MCV (r = 0.624). Among biochemical parameters, albumin demonstrated the largest effect (r = 0.596), followed by total bilirubin (r = 0.436) and SDMA (r = 0.419).

**Table 4:** The table shows the comparisons of biochemistry, blood gas and hematology parameters between the case (tooth resorption) and control (healthy) groups via the Mann-Whitney U test. The median, minimum and maximum values given reflect the center and variance of the distributions between both groups. The reference ranges show the normal ranges of the parameters. Effect sizes (r) with 95% confidence intervals are reported to quantify the magnitude and precision of the differences between the two groups.

	PARAMETERS	Control	TR		
		Median (Min–Max)	Median (Min–Max)	Reference Ranges	Effect Size (r) (95% CI)
**Biochemistry**	SDMA µg/dL	12.00 (9.00–14.00)	13.50 (10.00–24.00)	0.00–14.00	0.42 (0.115–0.725) *
	BUN mg/dL	24.65 (11.10–29.30)	18.25 (5.50–43.20)	4.70–34.00	0.27 (−0.049–0.597)
	Creatinine mg/dL	1.20 (0.90–2.60)	1.10 (0.80–1.80)	0.80–1.80	0.25 (−0.071–0.579)
	SGOT (AST) U/L	19.50 (11.00–34.00)	17.00 (11.00–57.00)	10.00–80.00	0.09 (−0.246–0.424)
	SGPT (ALT) U/L	50.00 (17.00–108.00)	35.50 (14.00–89.00)	10.00–80.00	0.39 (0.085–0.703) *
	Alkaline Phosphatase U/L	73.00 (27.00–251.00)	42.00 (17.00–108.00)	10.00–80.00	0.40 (0.088–0.706) *
	Amylase U/L	1126.50 (776.00–2078.00)	1521.00 (492.00–2326.00)	500.00–1800	0.13 (−0.199–0.467)
	Magnesium mg/dL	2.30 (1.80–2.70)	1.90 (1.60–2.70)	1.50–3.50	0.36 (0.044–0.672) *
	LDH U/L	204.50 (74.00–618.00)	169.50 (67.00–387.00)	75.00–490.0	0.03 (−0.311–0.361)
	Phosphorus mg/dL	5.55 (3.70–8.20)	4.90 (3.10–11.70)	1.80–6.40	0.31 (−0.010–0.630)
	Cholesterol mg/dL	174.50 (96.00–217.00)	153.00 (48.00–261.00)	90.00–205.0	0.17 (−0.160–0.502)
	Total Bilirubin mg/dL	1.35 (0.50–4.10)	0.80 (0.40–2.00)	0.10–0.60	0.44 (0.133–0.739) *
	Albumin g/dL	3.90 (3.70–4.40)	3.40 (2.30–4.30)	2.10–3.90	0.60 (0.326–0.866) *
	Calcium mg/dL	11.85 (10.70–12.80)	9.85 (8.30–12.80)	8.00–10.70	0.51 (0.223–0.801) *
	Triglyceride mg/dL	47.50 (29.00–63.00)	45.50 (19.00–156.00)	10.00–114.00	0.03 (−0.311–0.361)
	GGT U/L	3.00 (1.00–5.00)	2.00 (1.00–4.00)	1.00–10.00	0.31 (−0.006–0.632)
	Protein g/dL	7.80 (6.80–9.20)	8.25 (6.20–10.50)	5.40–7.80	0.17 (−0.167–0.497)
	CPK U/L	300.00 (94.00–749.00)	267.00 (136.00–1359.00)	50.00–450.0	0.01 (−0.325–0.347)
**Blood gas**	PH	7.34 (7.26–7.39)	7.36 (7.21–7.44)	7.36 (7.21–7.44)	0.27 (−0.053–0.595)
	pCO_2_ mmHg	36.45 (34.10–41.60)	34.20 (26.10–42.40)	34.20 (26.10–42.40)	0.36 (0.051–0.677) *
	pO_2_ mmHg	31.65 (25.80–42.40)	33.50 (26.80–53.50)	33.50 (26.80–53.50)	0.17 (−0.163–0.499)
	ctHb g/dL	15.90 (14.20–25.20)	13.65 (4.90–17.60)	13.65 (4.90–17.60)	0.60 (0.326–0.866) *
	HCT %	48.80 (43.50–77.10)	43.30 (15.00–53.80)	43.30 (15.00–53.80)	0.42 (0.121–0.729) *
	Potassium (K^+^) mmol/L	3.50 (3.20–4.50)	3.80 (3.30–4.80)	3.80 (3.30–4.80)	0.29 (−0.027–0.615)
	Sodium (Na^+^) mmol/L	164.50 (157.00–187.00)	160.50 (156.00–167.00)	160.50 (156.00–167.00)	0.44 (0.137–0.741) *
	Calcium (Ca) mmol/L	0.93 (0.70–1.30)	1.01 (0.83–1.43)	1.01 (0.83–1.43)	0.32 (0.006–0.642) *
	Chloride Cl^−^ mmol/L	123.50 (120.00–132.00)	124.00 (119.00–130.00)	124.00 (119.00–130.00)	0.05 (−0.288–0.384)
	Glucose mg/dL	80.50 (70.00–139.00)	89.50 (69.00–130.00)	89.50 (69.00–130.00)	0.22 (−0.107–0.549)
	Lactate mmol/L	2.60 (1.10–3.20)	2.05 (0.60–4.80)	2.05 (0.60–4.80)	0.02 (−0.319–0.353)
	Base(Ecf) mmol/L	−6.25 (−9.60–−3.70)	−7.10 (−12.50–−1.90)	−7.10 (−12.50–−1.90)	0.22 (−0.107–0.549)
	HCO_3_^−^ mmol/L	19.65 (17.10–21.70)	18.60 (14.20–22.60)	18.60 (14.20–22.60)	0.34 (0.022–0.654)
	Base(B) mmol/L	−5.55 (−8.80–−3.30)	−6.30 (−11.80–−1.20)	−6.30 (−11.80–−1.20)	0.22 (−0.107–0.549)
**Hematology**	WBC m/mm^3^	8.50 (4.95–17.00)	12.50 (3.15–44.00)	12.50 (3.15–44.00)	0.44 (0.137–0.741) *
	LYM %	38.75 (11.70–71.00)	46.60 (11.10–75.80)	7.00–60.00	0.12 (−0.214–0.454)
	MON %	0.85 (0.30–3.80)	7.15 (0.30–17.00)	1.00–9.00	0.57 (0.291–0.845) *
	GRA %	53.95(28.20–87.90)	47.85 (20.90–82.80)	47.85 (20.90–82.80)	0.18 (−0.152–0.510)
	LYM m/mm^3^	3.00 (0.70–6.60)	6.20 (0.54–33.40)	6.20 (0.54–33.40)	0.12 (−0.213–0.454) *
	MON m/mm^3^	0.85 (0.01–0.30)	0.90 (0.00–2.60)	0.90 (0.00–2.60)	0.57 (0.291–0.844) *
	GRA m/mm^3^	4.40 (2.60–10.30)	6.10 (1.90–24.80)	6.10 (1.90–24.80)	0.18 (−0.152–0.510)
	RBC m/mm^3^	9.83 (8.60–12.10)	10.20 (4.20–13.60)	10.20 (4.20–13.60)	0.10 (−0.240–0.430)
	MCV fl	38.80 (32.50–42.60)	45.90 (35.30–53.00)	38.00–54.00	0.62 (0.361–0.887) *
	HCT %	38.40 (32.80–47.30)	48.60 (16.70–64.20)	26.00–47.00	0.50 (0.210–0.792) *
	MCH pg	14.35 (12.50–16.20)	12.85 (11.40–17.80)	11.80–18.00	0.39 (0.076–0.696) *
	MCHC g/dL	37.35 (36.20–38.40)	28.20 (24.90–40.30)	29.00–36.00	0.67 (0.418–0.918) *
	RDW	18.05 (16.60–21.70)	12.55 (10.10–18.00)	12.55 (10.10–18.00)	0.71 (0.473–0.947) *
	HB g/dL	14.25 (12.50–17.20)	14.15 (5.10–18.20)	14.15 (5.10–18.20)	0.17 (−0.160–0.502)
	THR m/mm^3^	226.00 (142.00–302.00)	70.50 (16.00–398.00)	70.50 (16.00–398.00)	0.71 (0.473–0.947) *
	MPV fl	11.50 (7.90–14.00)	13.35 (10.60–14.50)	13.35 (10.60–14.50)	0.44 (0.137–0.741) *
	PCT %	2.40 (1.10–3.50)	0.10 (0.00–4.23)	0.10 (0.00–4.23)	0.70 (0.459–0.939) *
	PDW	14.40 (13.50–15.40)	0.00 (0.00–19.40)	12.00–17.50	0.60 (0.326–0.866) *

Note: Effect sizes (r) were calculated as |Z|/√N from Mann–Whitney U tests (N = 36: 24 TR, 12 Control). Interpretation: r < 0.10 = negligible effect; 0.10–0.30 = small effect; 0.30–0.50 = medium effect; ≥0.50 = large effect. Bold values indicate medium to large effect sizes (r ≥ 0.30), representing clinically meaningful differences. Asterisk (*) denotes statistical significance (*p* < 0.05).

#### 3.3.1. Biochemical and Blood Gas Findings

Statistical analysis of renal and enzymatic markers revealed that serum SDMA concentrations were significantly higher in the TR group compared to the control group (*p* = 0.011). Significant elevations were also observed in alkaline phosphatase (*p* = 0.016), alanine aminotransferase (ALT; *p* = 0.016), and magnesium (*p* = 0.032) levels. Conversely, certain parameters were significantly decreased in cats with TR, most notably albumin (*p* < 0.001) and calcium (*p* < 0.001) concentrations. Regarding blood gas analysis, significant differences were detected between the groups for pCO_2_ (*p* = 0.032), total hemoglobin (*p* < 0.001), and hematocrit (*p* < 0.001). Furthermore, sodium (*p* = 0.011) and ionized calcium (*p* = 0.038) levels showed statistically significant variations, further characterizing the systemic biochemical alterations associated with tooth resorption.

#### 3.3.2. Hematological Findings

Analysis of the leukogram parameters indicated a significant inflammatory state in cats with TR, as evidenced by markedly higher white blood cell counts (*p* = 0.007), absolute monocyte counts (*p* < 0.001), and lymphocyte counts (*p* = 0.038) compared to the control group. Significant alterations were also observed in the erythrogram profiles, including variations in red blood cell indices such as mean corpuscular volume (*p* < 0.001), mean corpuscular hemoglobin (*p* = 0.024), and mean corpuscular hemoglobin concentration (MCHC; *p* < 0.001). Furthermore, platelet dynamics were significantly affected in the TR group, which exhibited lower overall platelet counts (*p* < 0.001) alongside significant differences in mean platelet volume (*p* = 0.014) and plateletcrit (*p* < 0.001). These hematological shifts collectively reflect the systemic impact and the chronic inflammatory nature of feline tooth resorption.

### 3.4. Multivariable Analysis and Independent Predictors

A binomial logistic regression analysis was performed to identify independent predictors for the presence of TR. The model, incorporating SDMA, creatinine, age, and sex, demonstrated a robust fit (Nagelkerke R^2^ = 0.705). The Hosmer–Lemeshow goodness-of-fit test indicated adequate model fit (χ^2^ = 2.248, df = 7, *p* = 0.945), with no significant departure from expected values across deciles of predicted probability. The model correctly classified 83.1% of cases overall (sensitivity: 78.9%; specificity: 86.8%), with an events-per-variable ratio of 6 (24 events/4 predictors), which is considered adequate for stable parameter estimation. The logistic regression equation was as follows: logit(P) = −10.429 + 1.269(SDMA) − 3.915(Creatinine) + 0.046(Age) + 1.665(Sex) where P is the probability of tooth resorption, SDMA is expressed in μg/dL, creatinine in mg/dL, age in years, and sex is coded as 0 (female) and 1 (male). The key findings, detailed in Table 5, are as follows:

- SDMA: SDMA was identified as a strong and independent predictor of TR (B = 1.269, SE = 0.504, Wald = 6.345, *p* = 0.012). Each 1 μg/dL increase in serum SDMA was associated with a 3.6-fold increase in the odds of tooth resorption (OR = 3.556, 95% CI: 1.324–9.548), indicating a clinically meaningful positive association between early renal biomarker elevation and feline dental pathology.
- Creatinine: Creatinine showed a statistically significant negative association with TR (B = −3.915, SE = 1.935, Wald = 4.094, *p* = 0.043; OR = 0.020, 95% CI: 0.000–0.885). The negative regression coefficient and OR below 1.0 indicate that higher serum creatinine concentrations were associated with decreased odds of tooth resorption; specifically, each 1 mg/dL increase in creatinine was associated with a 98% reduction in the odds of TR. This counterintuitive finding warrants cautious interpretation and is discussed in detail below.
- Model Diagnostics: Multicollinearity diagnostics revealed acceptable VIF values for all predictors: SDMA (VIF = 2.875, Tolerance = 0.348), creatinine (VIF = 2.875, Tolerance = 0.348), age (VIF = 1.028, Tolerance = 0.973), and sex (VIF = 1.007, Tolerance = 0.993). The identical VIF values for SDMA and creatinine reflect their expected correlation as renal function biomarkers but remained well below the threshold of concern.

Post hoc power analysis demonstrated that the logistic regression model achieved adequate power for the two significant predictors, SDMA (1 − β = 0.817) and creatinine (1 − β = 0.999), confirming that both associations are robust and not attributable to Type II error. In univariate analysis, SDMA approached but did not fully meet the conventional power threshold (1 − β = 0.70), though its consistent significance across both univariate and multivariable analyses strengthens confidence in this finding. As expected, non-significant predictors demonstrated markedly lower power for age (1 − β = 0.051), whereas sex showed high power (1 − β = 0.938) despite non-significance (*p* = 0.276), indicating adequate sensitivity and confirming a genuine lack of association rather than Type II error. The chi-square test for sex distribution showed borderline power (1 − β = 0.682), suggesting that group matching for sex was adequate but not optimally powered.

## 4. Discussion

The present study investigated the association between tooth resorption (TR) and selected renal and systemic biomarkers in cats, with a primary emphasis on SDMA. To the authors’ knowledge, this is one of the few clinical studies specifically evaluating the clinical association of SDMA with resorptive lesions. Our findings demonstrated that serum SDMA concentrations were significantly higher in the TR group and, more importantly, each unit increase in SDMA was independently associated with a 3.5-fold increase in the likelihood of TR presence. While previous studies have highlighted the link between periodontal disease and chronic kidney disease (CKD) often referred to as the “oral-renal axis” the specific role of SDMA as an early marker in the context of TR has remained largely unexplored [22,23]. Unlike creatinine, which is heavily influenced by muscle mass and often remains within reference ranges until significant nephron loss occurs, SDMA has been shown to detect glomerular filtration rate (GFR) reduction much earlier in feline patients [24,25].

All cats included in the study underwent the same standardized anesthetic protocol prior to radiographic examination and blood sampling. While this approach minimized procedural variability between groups, it is important to acknowledge that anesthetic agents such as medetomidine, butorphanol, and propofol may influence renal perfusion and glomerular filtration rate. Alpha-2 adrenergic agonists, including medetomidine, have been documented to reduce renal blood flow and GFR in feline patients through systemic vasoconstriction [26,27]. However, recent studies suggest that serum SDMA concentrations are less susceptible to transient physiological fluctuations compared to traditional markers, providing a more stable reflection of renal function even under brief periods of altered hemodynamics [28]. Since the anesthetic protocol was strictly standardized across both the TR and control groups, any potential drug-induced alterations would have affected both cohorts equally, thereby maintaining the validity of the observed inter-group differences in SDMA and other biomarkers.

In the present study, tooth resorption was diagnosed in 66.7% of the evaluated cats, a finding that aligns with the upper limit of the prevalence range (20–75%) reported in previous global studies [29,30]. Although prevalence estimation was not a primary objective, this high frequency reinforces the clinical significance of TR in the domestic cat population. It should be noted, however, that the high prevalence observed may reflect the specific nature of our study population, which consisted of cats presented to a tertiary referral hospital, a setting that may naturally include a higher proportion of cats with underlying dental pathology compared to the general feline population. All TR diagnoses were confirmed radiographically, supporting the diagnostic approach advocated by Eriksson et al. [31], who demonstrated that dental radiography has substantially higher sensitivity for detecting resorptive lesions compared to clinical examination alone. Our findings further emphasize that intraoral radiography is an indispensable tool not only for accurate identification but also for the precise staging and treatment planning of TR, as many lesions remain hidden beneath the gingival margin or involve the roots without overt clinical signs.

Age is a well-recognized risk factor for the development of tooth resorption in cats, with both the number of affected teeth and lesion severity typically increasing with advancing age [1,32]. In our study, although the TR group had a slightly higher median age, statistical analysis confirmed that the groups were well-balanced, and no significant age discrepancy existed (*p* > 0.05). Furthermore, our multivariate logistic regression model demonstrated that age was not an independent predictor for the presence of TR in this study population (*p* = 0.189). This finding is particularly important as it suggests that the observed elevations in serum SDMA and other biomarkers are more closely associated with the presence of resorptive lesions themselves rather than being merely a reflection of age-related renal decline. By achieving age-homogeneity between the TR and control groups, we have minimized the potential confounding effect of age, thereby strengthening the association found between TR and altered renal/systemic parameters.

The anatomical distribution of affected teeth in the present study is consistent with previous reports identifying the mandibular third premolars and first molars as the most frequently involved teeth [29,33]. Our finding that the mandibular right first molar (tooth 409) and the maxillary right fourth premolar (tooth 108) exhibited the highest frequency of lesions corroborates the well-documented patterns of TR localization. Furthermore, a significant association between TR stage and TR type was identified, corroborating findings reported by Whyte et al. [34]. Specifically, early-stage lesions (Stages 1 and 2) were predominantly associated with Type 1 (inflammatory) resorption, whereas advanced stages (Stages 3 to 5) were more frequently linked to Type 3 (replacement) resorption. These results support the clinical utility of the AVDC [6] classification system and highlight the paramount importance of radiographic assessment for accurate lesion characterization and surgical decision-making.

In the present study, serum SDMA concentrations were significantly higher in cats with TR compared to the control group (*p* = 0.011), with a medium effect size (r = 0.42) and adequate power in multivariable analysis (1-β = 0.817), supporting the robustness of this association, whereas serum creatinine and blood urea nitrogen concentrations did not exhibit significant differences.

The negative association between serum creatinine and tooth resorption observed in the multivariable model (OR = 0.020, 95% CI: 0.000–0.885, *p* = 0.043) appears counterintuitive and warrants careful interpretation. Several explanations may account for this finding. First, it may represent a statistical artifact arising from the relatively small sample size and low number of events, which can produce wide confidence intervals and unstable coefficient estimates in logistic regression. Second, the simultaneous inclusion of SDMA and creatinine-correlated renal biomarkers (VIF = 2.875 for both) may have introduced residual collinearity effects that distorted individual coefficient estimates, even though VIF values remained within acceptable limits. Third, this pattern may reflect a genuine biological phenomenon in which cats with higher SDMA but lower creatinine represents an early subclinical renal dysfunction phenotype, wherein glomerular filtration is impaired but muscle mass, a key determinant of creatinine production, remains relatively preserved [24,35]. Notably, the very high power of the creatinine coefficient in the logistic regression (1 − β = 0.999) indicates that this finding is unlikely to be a Type II error; the association, however paradoxical, appears statistically robust and warrants further investigation in larger, prospective studies.

The moderate VIF values shared by SDMA and creatinine (VIF = 2.875) are biologically expected given that both markers rise with declining glomerular filtration rate [24,35]. The retention of both variables in the final model was justified by their kinetically distinct behavior: SDMA is detectable earlier in the course of renal dysfunction and is less influenced by lean muscle mass than creatinine, making their combined inclusion clinically informative rather than redundant.

SDMA has been widely recognized as a more sensitive indicator of reduced glomerular filtration rate (GFR) than creatinine, particularly in the early stages of renal dysfunction, as it is less influenced by extra-renal factors such as muscle mass [24,25]. Crucially, our multivariate logistic regression analysis identified SDMA as a strong independent predictor of TR presence, with each unit increase in SDMA associated with a 3.5-fold increase in the likelihood of having resorptive lesions. Since the age distribution between the TR and control groups was statistically homogeneous (*p* > 0.05), this association cannot be attributed solely to age-related renal decline. Instead, it suggests a potential ‘oral-renal axis’ where chronic inflammatory processes associated with TR may lead to early subclinical renal impairment or where systemic SDMA metabolism is altered by the chronic inflammatory milieu [28]. The multivariable logistic regression model employed in our study was adjusted for age, sex, and other potential clinical confounders. While the general link between periodontal inflammation and renal biomarkers in cats is recognized, our model identified SDMA as a significant clinical marker for the presence of TR independent of these factors. This suggests that resorptive lesions do not merely coexist with periodontal disease but may represent a distinct pathological entity capable of contributing to the systemic inflammatory load and subsequent renal biomarker alterations. While the association between SDMA and TR is primarily attributed to early-stage renal impairment secondary to chronic systemic inflammation, alternative pathophysiological mechanisms warrant consideration. It is possible that the chronic inflammatory milieu in TR patients induces systemic endothelial dysfunction or alterations in arginine metabolism, both of which are closely linked to the synthesis and clearance of methylarginines. Specifically, the persistent activation of inflammatory pathways may influence the activity of protein arginine methyltransferases (PRMTs), leading to elevated SDMA concentrations independent of traditional GFR decline. Incorporating these non-renal influences provides a more comprehensive perspective on the complex interplay between oral pathology and systemic biomarker dynamics.

Albumin is a major plasma protein and a negative acute-phase reactant, with concentrations known to decrease during chronic inflammatory states due to the down-regulation of hepatic synthesis by pro-inflammatory cytokines [36,37]. In the present study, serum albumin concentrations were significantly lower in cats with TR (*p* < 0.001), while total protein levels remained comparable between groups. Previous studies have reported inconsistent findings regarding albumin and albumin-to-globulin (A:G) ratios in feline TR patients; while some identified no significant changes, others noted a similar downward trend in chronic cases [29,34]. Although hypoalbuminemia is a non-specific finding influenced by nutrition, hepatic function, and systemic conditions, its subclinical decrease in our TR cohort likely reflects the persistent systemic inflammatory response elicited by chronic resorptive lesions. This finding, coupled with the observed leukocytosis and monocytosis, further supports the hypothesis that feline TR is not merely a localized oral condition but a disease with significant systemic implications.

In the present study, several hematological parameters, including white blood cell (WBC), monocyte, and lymphocyte counts, were significantly higher in cats with TR compared to the control group (*p* < 0.05). It is noteworthy that although these parameters showed statistically significant differences, most values remained within their respective clinical reference intervals. In the field of clinical pathology, such ‘within-range’ shifts are often interpreted as subclinical alterations rather than overt pathological crises. The tendency of inflammatory markers to cluster toward the upper limit of the reference range in cats with TR suggests a low-grade, chronic systemic inflammatory response elicited by persistent odontoclastic activity. Specifically, the low grade monocytosis observed in the TR cohort (*p* < 0.001) likely reflects a persistent innate immune response to the ongoing hard tissue destruction and secondary bacterial involvement associated with resorptive lesions. While leukocytosis and monocytosis can be influenced by stress or non-specific factors [34], their concurrence with significantly lower albumin levels in our study supports the presence of a sustained systemic inflammatory milieu. These findings corroborate the hypothesis that TR-associated inflammation is not confined to the oral cavity but triggers measurable systemic hematological alterations, which may further contribute to the development of subclinical renal dysfunction as indicated by elevated SDMA levels.

Beyond the primary inflammatory markers, the significant alterations in erythrocyte indices (MCHC and RDW) and the marked decrease in platelet parameters (*p* < 0.001) in the TR group suggest a more complex systemic involvement. The lower MCHC levels may reflect the early stages of ‘anemia of chronic disease,’ where persistent oral inflammation interferes with iron metabolism and erythropoiesis [36]. Furthermore, the significant decline in platelet count and plateletcrit suggests a state of chronic platelet activation or consumption at the sites of active dental resorption. The negative association between serum creatinine and tooth resorption observed in the multivariable model (OR = 0.020, 95% CI: 0.000–0.885, *p* = 0.043) appears counterintuitive and warrants careful interpretation. Several explanations may account for this finding. First, it may represent a statistical artifact arising from the relatively small sample size and low number of events, which can produce wide confidence intervals and unstable coefficient estimates in logistic regression. Second, the simultaneous inclusion of SDMA and creatinine-correlated renal biomarkers (VIF = 2.875 for both) may have introduced residual collinearity effects that distorted individual coefficient estimates, even though VIF values remained within acceptable limits. Third, this pattern may reflect a genuine biological phenomenon: cats with higher SDMA but lower creatinine may represent an early subclinical renal dysfunction phenotype in which glomerular filtration is impaired but muscle mass, a key determinant of creatinine production, remains relatively preserved [24,35]. In this context, cats with advanced TR may experience chronic oral pain and secondary inappetence, leading to reduced body condition and loss of skeletal muscle mass, which would further lower serum creatinine independently of GFR status. This mechanism underscores the clinical utility of SDMA in this population: as a biomarker independent of muscle mass, SDMA provides a more consistent reflection of renal function in cats where chronic disease may have compromised body condition [24]. Notably, the very high power of the creatinine coefficient in the logistic regression (1 − β = 0.999) indicates that this finding is unlikely to represent a Type II error; the association, however paradoxical, appears statistically robust and warrants further investigation in larger, prospective studies. The moderate and identical VIF values for both SDMA and creatinine (VIF = 2.875) are biologically expected, as both markers rise with declining glomerular filtration rate [24,35]; however, their kinetically distinct behavior SDMA being detectable earlier and unaffected by lean muscle mass justified their combined inclusion in the model as clinically complementary rather than redundant predictors. These findings collectively reinforce that TR triggers a cascade of subclinical hematological and metabolic shifts that extend far beyond the oral cavity. From a clinical perspective, these findings suggest that SDMA might be considered a helpful ancillary biomarker during the comprehensive evaluation of cats diagnosed with TR. In patients presenting with multiple or radiographically advanced resorptive lesions, performing a renal screening that includes SDMA could be beneficial, even when creatinine concentrations remain within established reference intervals. For cases where elevated SDMA is identified alongside TR, periodic monitoring tailored to the patient’s overall clinical status might be considered as a potential approach to assess renal stability over time. Furthermore, the possibility that SDMA levels may fluctuate in response to the oral inflammatory load suggests that integrating dental health assessments into the management of early-stage renal dysfunction could be a prudent clinical consideration.

In summary, this study identified significant associations between tooth resorption and selected biochemical and hematological parameters, most notably serum SDMA and albumin. Our findings demonstrate that SDMA is a potent independent factor associated with the presence of TR, with a 3.5-fold increase in risk for each unit elevation, even when adjusted for age and sex. The prospective design of this study, utilizing a carefully selected age-homogeneous control group and robust multivariate statistical modeling, strengthens the validity of the observed ‘oral-renal’ associations. These results suggest that feline TR is associated with a state of low-grade systemic inflammation and subclinical renal alterations. Future longitudinal studies are warranted to further elucidate the temporal relationship between the progression of resorptive lesions and the decline in glomerular filtration rate, potentially establishing SDMA as a routine monitoring tool for cats with feline dental resorptive lesions.

The present study has several limitations that should be considered. While we achieved statistical homogeneity for age and sex between the TR and control groups (*p* > 0.05) and utilized multivariate logistic regression to control for these variables, the prospective nature of the study may still involve unmeasured confounding factors. However, the nature of this observational study does not allow for a definitive establishment of a causal relationship between tooth resorption and elevated SDMA levels. While the association is statistically significant, it remains unclear whether TR-induced systemic inflammation directly impairs renal function or if both conditions share common, yet unidentified, pathophysiological pathways. Therefore, these findings should be interpreted as a potential clinical correlation rather than a direct causative link, necessitating longitudinal studies to clarify the temporal sequence of these alterations. Despite the prospective nature of this study, a definitive causal relationship specifically whether elevated SDMA precedes TR or vice versa cannot be firmly established within this observational framework. However, the use of a meticulously age-balanced control group and the finding that each unit increase in SDMA is associated with a 3.5-fold increase in the likelihood of TR suggest a non-coincidental association. These results provide significant support for the ‘oral-renal axis’ hypothesis and establish a robust framework for future longitudinal prospective investigations. A significant limitation is the absence of urine specific gravity (USG) measurements, which are recommended by the International Renal Interest Society (IRIS) guidelines for a comprehensive assessment of renal function. Due to technical and financial constraints, urinalysis was not performed; therefore, the renal alterations suggested by elevated SDMA levels could not be further characterized through USG or proteinuria assessment. Additionally, while the standardized anesthetic protocol minimized procedural variability, any potential transient influence of agents like medetomidine on GFR was avoided, as all blood samples were obtained prior to the administration of any anesthetic or sedative agents. Furthermore, a notable sex imbalance was present in the control group, primarily due to the recruitment of subjects from elective orchiectomy cases. While sex was included in the multivariate logistic regression model to adjust for its potential confounding effects, the skewed distribution remains a methodological limitation that may influence the robustness of the sex-related findings. Finally, although our multivariate model demonstrated robust associations, the relatively small sample size (n:36) and the number of parameters evaluated necessitate caution. Specifically, the borderline events-per-variable (EPV) ratio in the logistic regression model and the absence of adjustment for multiple comparisons may increase the risk of Type I errors. Post hoc power analysis confirmed that the study achieved adequate power for the primary outcome: SDMA in logistic regression (1 − β = 0.817). However, univariate power for SDMA was borderline (1 − β = 0.70), and several comparisons with small-to-medium effect sizes, were underpowered. The use of effect sizes alongside *p*-values throughout this study was intended to provide a more complete picture of clinical relevance, independent of sample size constraints [38]. Additionally, tooth resorption was analyzed as a binary outcome (presence/absence), which may oversimplify the heterogeneous nature of this condition. The affected cats in the present study showed considerable variation in lesion number, stage, and anatomical distribution, and this simplification may have obscured potential dose–response relationships between lesion burden and the magnitude of systemic biomarker alterations. Future studies incorporating ordinal or continuous measures of disease severity such as total number of affected teeth or cumulative lesion staging scores would provide greater in-sight into these relationships. These findings should be considered hypothesis-generating, and larger, prospective longitudinal studies are required to confirm the independent diagnostic associations of SDMA in feline tooth resorption. While the cross-sectional nature of this study precludes establishing a definitive predictive or prognostic role for SDMA in TR progression, our findings demonstrate a robust clinical association that warrants further longitudinal investigation. Therefore, these findings should be strictly interpreted within a hypothesis-generating and exploratory framework rather than as confirmatory evidence.

## 5. Conclusions

The present study demonstrates a significant association between feline tooth resorption (TR) and altered systemic biomarkers, most notably serum SDMA and albumin. Our multivariate analysis confirmed that SDMA is independently associated with TR presence, independent of age and sex, with each unit increase in SDMA concentration associated with a 3.5-fold increase in the likelihood of having resorptive lesions. These results suggest that feline TR is not merely a localized oral condition but is associated with a state of low-grade systemic inflammation and subclinical renal alterations. While serum albumin levels are lower in affected cats, reflecting a sustained negative acute-phase response, SDMA emerges as a promising biomarker for monitoring the ‘oral-renal axis’ in feline patients. However, while SDMA shows promise in the clinical assessment of cats with TR, its routine clinical application should currently be regarded as a potential future perspective rather than a definitive recommendation, pending confirmation from longitudinal studies. By shifting the perspective of TR from a purely dental concern to a potential systemic involvement, this study advocates for a more holistic diagnostic approach in feline medicine. Future prospective longitudinal studies are warranted to further elucidate the causal link and investigate whether timely dental intervention can mitigate the progression of these systemic alterations.

## Figures and Tables

**Table 1:** Individual characteristics of cats included in the present study.

Variable		TR Group (24 Cats)	Control (12 Cats)
Median Age (year)		3.33	2.83
Sex	Male	12	11
	Female	12	1
Breed	Domestic short hair	13	4
	Persian	1	-
	Siamese	1	1
	British Short hair	4	2
	Russian Blue	1	-
	Van Cat	2	-
	Bombay	1	-
	Scottish Fold	1	4
	Chinchilla	-	1
Feeding Type	Dry Kibble	7	5
	Dry kibble, moist food	3	1
	Mix Feeding	14	6

**Table 2:** Classification of the total number of teeth affected by tooth resorption in the TR group, expressed as a percentage according to anatomical numbering.

Modified Triadan Tooth Numbering	TR Affected (118 Teeth)
102	118/1 (0.84%)
104	118/7 (5.93%)
201	118/2 (1.69%)
202	118/2 (1.69%)
204	118/8 (6.78%)
304	118/7 (5.93%)
404	118/7 (5.93%)
107	118/4 (3.38%)
108	118/11 (9.32%)
206	118/1 (0.84%)
207	118/4 (3.38%)
208	118/9 (7.62%)
307	118/8 (6.78%)
308	118/8 (6.78%)
407	118/7 (5.93%)
408	118/7 (5.93%)
309	118/10 (8.47%)
409	118/16 (13.56%)

Note: Data are reported as counts/N(%).

**Table 3:** Relationship between the TR stage and TR type considering the number of resorbed teeth.

TR Type	TR Stage						
	1 (*n* = 3)	2 (*n* = 36)	3 (*n* = 2)	4a (*n* = 2)	4b (*n* = 5)	4c (*n* = 72)	5 (*n* = 8)
1	1/3 ^a,b^ (%33.33)	12/26 ^a^ (%46.20)	-	-	1/4 ^a^ (%25)	-	-
2	2/3 ^b^ (%66.67)	10/26 ^a^ (%38.50)	-	-	1/4 ^a^ (%25)	4/63 ^a^ (%6.30)	-
3	-	4/26 ^b^ (%15.4)	2/2 ^a^ (%100)	2/2 ^a^ (%100)	2/4 ^a^ (%50)	59/63 ^b^ (%93.70)	6/6 ^a^ (%100)

Note: Values with different superscripts within a row are significantly different at *p* < 0.05.

**Table 5:** Final model for binary logistic regression, showing the associations of TR (tooth resorption) with SDMA, creatinine, age, sex.

Variable	B	S.E.	Wald	df	OR (95% CI)	*p*-Value
SDMA	1.269	0.504	6.345	1	3.556 (1.325–9.543)	0.012 *
Creatinine (mg/dL)	−3.915	1.935	4.094	1	0.020 (0.000–0.885)	0.043 *
Age	0.046	0.035	1.727	1	1.047 (0.978–1.121)	0.189
Sex	1.665	1.527	1.189	1	5.285 (0.265–105.353)	0.276
Constant	−10.429	5.351	3.799	1	0.000	0.051 *

B: regression coefficient; SE: standard error; df: degrees of freedom; OR: Odds ratio. * indicates statistical significance.

## Data Availability

The data presented in this study are available on request from the corresponding author. The data are not publicly available due to ethical and privacy restrictions, as the study involves clinical records of privately owned animals. Sharing the full raw dataset publicly could potentially compromise the confidentiality and privacy of the animal owners. Additionally, the data are subject to institutional regulations regarding the protection of clinical patient information.

## Abbreviations

The following abbreviations are used in this manuscript:

TR	Tooth Resorption
SDMA	Symmetric Dimethylarginine
CKD	Chronic Kidney Disease
GFR	Glomerular Filtration Rate
AVDC	American Veterinary Dental College
IRIS	International Renal Interest Society
BUN	Blood Urea Nitrogen
ALT	Alanine Aminotransferase
MCHC	Mean Corpuscular Hemoglobin Concentration
ctHb	Total Hemoglobin
pCO2	Partial Pressure of Carbon Dioxide
